# Breast Implant Illness: A Case Series

**DOI:** 10.7759/cureus.23680

**Published:** 2022-03-31

**Authors:** Peter M Habib, Thomas Serena, Amy Derosa

**Affiliations:** 1 Department of Surgery, Beaumont Hospital-Farmington Hills, Farmington Hills, USA

**Keywords:** implant-based breast augmentation, breast implant complications, en bloc breast implant removal, implant removal, breast implant illness

## Abstract

Since the advent of breast implants, there has been unprecedented controversy and FDA bands regarding their safety. There has been a demonstrated link with certain types of lymphoma, autoimmune disorders, and systemic illness associated with breast implants. A significant population of women currently pursue bilateral breast implant removal in hopes to alleviate a constellation of symptoms anecdotally known as “breast implant illness”. This is not yet an accepted clinical entity due to the lack of sound literature on the subject. Common presenting symptoms include fatigue, anxiety, chronic pain, endocrine, autonomic, and peripheral nervous system dysfunction. Currently, there is no standard of care or guideline for treating women experiencing such symptoms. The current literature regarding breast implant illness has been widely observational and descriptive. With over four million women across the globe with augmented breasts, the potential impact of this research is great. This paper presents three patients believed to be suffering from breast implant illness, who after en-bloc resection, experienced resolution of their symptoms.

## Introduction

The invention and progression of breast augmentation have not come without controversy. As with any surgical procedure, the risk is inherent. There has been demonstrated linking breast implants with lymphoma, autoimmune disorders, and systemic illness [1-3]. At the onset of the 21^st^ century, anecdotal data began to surface describing a disease process related to implants. Commonly known as “breast implant illness”, this pathology is characterized by symptoms such as fatigue, anxiety, chronic pain, and exacerbation of endocrine, autonomic, and peripheral nervous system dysfunction. Furthermore, somatic dysfunction may play a significant role, manifesting as the impaired or altered functions of related components of the somatic (body framework) system: skeletal, arthrodial, and myofascial structures and related vascular, lymphatic, and neural elements.

A significant population of women is now pursuing the removal of their breast implants to cure their implant-associated symptoms. No standard of care nor guideline currently exists for treating women experiencing such symptoms. This research seeks to supplement the literature base and eventually provide a framework for best practice guidelines for treating women with breast implant illness. This paper presents three patients with varying symptomatology requesting removal of their implants in hopes of a cure.

## Case presentation

Case one: 49-year-old female

The patient underwent a saline breast augmentation via inframammary fold (IMF) incision in May of 1994 for cosmetic reasons. The patient first reported symptoms three years post-implantation consisting primarily of nonspecific chest pain. Additional reported symptoms included fatigue, tinnitus, daily headache, dizziness, vision changes, dyspnea, palpitations, constipation, easy bruising, myalgia, arthralgia, edema, anxiety, and depression. Preoperatively, the patient required Holter monitoring for continuous palpitations. Labs and imaging workup were unremarkable. Her past medical history was significant for untreated rheumatoid arthritis, hypothyroidism, and premature ventricular contractions. She had no known drug allergies. Family and social history were non-contributory. Pre-operative physical exam revealed grade II capsular contracture, an inframammary fold (IMF) scar, and bilateral breast ptosis over implants.

The patient was brought to the operative theater for bilateral breast implant removal with en-bloc capsulectomy. After heart and lung examinations were complete, general anesthesia and full muscle paralysis were administered by the department of anesthesia. The patient was placed in a supine position, and the areas of the chest and upper abdomen were trimmed, cleaned, and prepped with betadine. These areas were draped in a sterile fashion. An incision was made along the previous scar. Dissection proceeded through the subcutaneous tissue using sharp dissection and electrocautery until the implant capsule was encountered. The capsule was found to be submuscular. Implant and capsule were removed from breast pocket en-bloc. The breast cavity was copiously irrigated with a triple antibiotic (Adam’s) solution. Hemostasis was achieved using electrocautery. Jackson-Pratt (JP) drain was placed within the pocket and sutured to the chest wall as it exited laterally with a 3-0 nylon stitch. The muscle was reapproximated to the chest wall with 0 Vicryl. Skin edges were sharply debrided with gorney scissors as needed and approximated with 3-0 Vicryl followed by a running 4-0 Monocryl suture. The procedure was repeated on the contralateral breast. Foam tape and a surgical support bra were placed. Good capillary refill of the nipple and skin flaps was confirmed. Patients were all discharged on cephalexin or clindamycin while drains were in place. Drains were removed when output was less than 30 milliliters in 24 hours. 

One week postoperatively, the patient reported complete resolution of her symptoms despite no changes in her home medications. Beginning on a postoperative day one, she noticed a difference in her skin complexion and overall energy. The patient denies palpitations since surgery. She endorsed better sleep and a complete resolution of previously described diffuse myalgias and arthralgias. On physical examination, the patient’s incisions were clean dry, and intact. The flaps were pink without ecchymosis and had excellent breast shape. On a follow-up phone call six months postoperatively, the patient was very pleased with her results and reported no return of previously described symptoms.

Case two: 50-year-old female

This patient underwent saline breast augmentation in 2003 for cosmesis. Her symptoms presented two years after implantation and consisted of inflamed and painful joints in her hands, chronic dizziness, vision changes, ‘brain fog’, dyspnea, urinary urgency, dysuria, bladder spasm, yeast infections, cold and heat intolerance, decreased libido, easy bruising, debilitating panic attacks, palpitations, and repeated upper respiratory infections. She was seen by neurology and a chiropractor for vision changes. Her past medical history is significant for anxiety, chronic yeast infections, Sjogren’s Syndrome diagnosed six months post-implant, and Anti-Thrombin III deficiency. She has no known drug allergies. She does not take any prescription medications. Family history was non-contributory. Social history is significant for a half a pack per day of smoking. Pre-operative physical examination showed grade I capsular contracture with Le Jour Hockey stick incisions. She underwent bilateral breast implant removal with en-bloc capsulectomy in the standardized fashion presented in patient one.

One week postoperatively, the patient reported her vision changes improved rapidly after surgery. She also expressed that her facial complexion improved immediately. On post-operative physical examination, the incisions were clean, dry, and intact. Both flaps demonstrated a good capillary refill without ecchymosis. On a follow-up phone call two months later, the patient reported her vision had continued to improve. Additionally, the frequency of her joint pain continued to decrease in frequency.

Case three: 40-year-old female

This patient underwent silicone breast augmentation in 2008 for cosmetic reasons. She was initially with smooth implants; however, they were hypermobile, causing her discomfort, and she switched to textured silicone implants. She reported her symptoms began one month following the final implantation in 2008. It began with fatigue, and as months progressed, she began experiencing changes in her menses, constipation, palpitations, and dry skin. She began to feel more depressed and low energy. She was constantly thwarted with left-sided headaches. Other neurological symptoms manifested with left upper extremity numbness. This made her more anxious, and she started to have panic attacks. She was evaluated in the office early in 2020. On examination, the patient had double D-sized breasts with grade 1 capsular contraction. The patient reported she had gone from a B cup to a DD. Past medical history was significant for depression and anxiety, for which she was treated with Zoloft. Surgical history only noted breast augmentation and revision of initial implant. Medications in addition to sertraline included diphenhydramine and ibuprofen on occasion. She has a penicillin allergy. Both of her parents have passed. Their mother died after a cerebrovascular accident and her father after myocardial infarction. She underwent bilateral explantation on January 29, 2020. On operative evaluation, the right implant was a silicone textured implant intact within the capsule; however, the left implant was noted to be ruptured. On postoperative evaluation at three weeks, the patient’s anxiety and panic attacks had ceased. She completely denied the numbness she previously had in the left upper extremity. She had no headaches or palpitations and also noticed her skin tone and the whites of her eyes had cleared compared to before surgery. Follow up phone call approximately eight months later confirms no recurrence of panic attacks. She has since lost 15 pounds and has no complaints or reservations about her decision.

## Discussion

Breast implant illness is an anecdotal clinical entity describing a variety of symptoms thought to be a direct result of the presence of breast implants. The most common presenting symptoms are fatigue, brain fog, chronic pain, anxiety, hair loss, and exacerbations of autoimmune, endocrine, and neurological diseases [1]. Women suffering from this clinical entity often present after developing diverse symptoms finally attributed to their implants after multiple futile evaluations by subspecialists. Formal recommendations do not exist for the treatment of women with complaints believed to be related to their implants. After a thorough analysis of available research and anecdotal experiences, the writers now seek to be the first to truly define breast implant illness as a true pathology.

We have demonstrated success in treating these patients using a standardized surgical approach consisting of en-bloc resection, including capsulectomy. We include capsulectomy due to the concern that the retained capsule could further potentiate what may be a systemic inflammatory reaction. There is no literature on en-bloc resection as a treatment for breast implant syndrome; however, a single study in the Netherlands showed that 69% of women with explantation of implants showed complete symptom resolution [4]. However, this reported cohort did not comment on the procedure performed [3].

**Figure 1:**
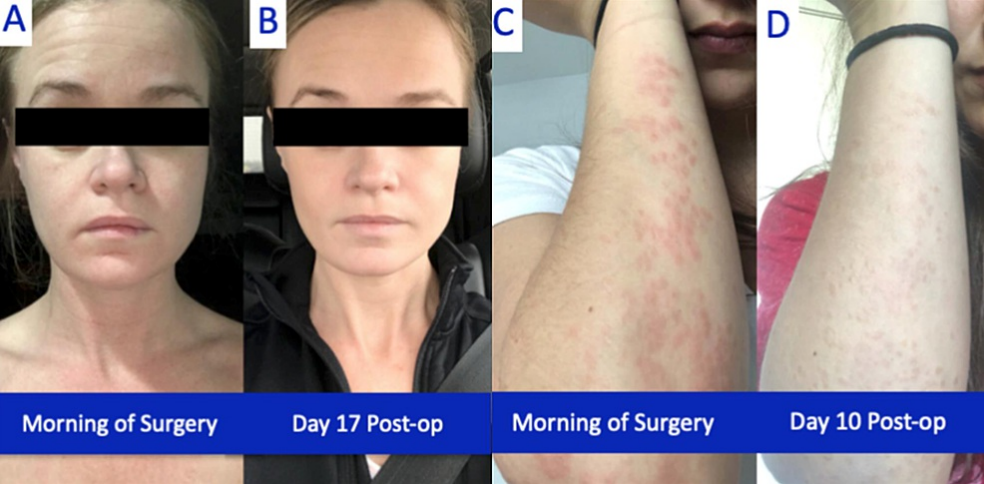
Pre- and post-operative photographs of two different sample patients and their dermatologic symptom improvement. (A) and (B) demonstrate facial erythema decreasing and skin clearing. (C) and (D) note a decrease in the visibility of the forearm erythema and the raised nature of the patient’s rash post-operatively.

The writers theorize improvement after en-bloc resection may relate to the removal of a nidus for systemic inflammation. Whether this relates to the new onset of disease and disorder or potentiation of underlying systemic illness, the operation serves in a sense as source control of the precipitator of illness. In addition, altered lymphatic drainage by a foreign body impeding on the thoracic inlet may add a physical or somatic component to the disease process. This is evidenced by conjunctival injection and changes in skin complexion, which resolve immediately the following explantation. Other noticeable results are various rashes that seem to drastically improve following surgery (Figure 1). With over four million women across the globe with augmented breasts, this research has a large potential impact [5]. At this time, available anecdotal data demands higher quality research to further define this entity and the potential benefits of surgical treatment.

**Figure 2:**
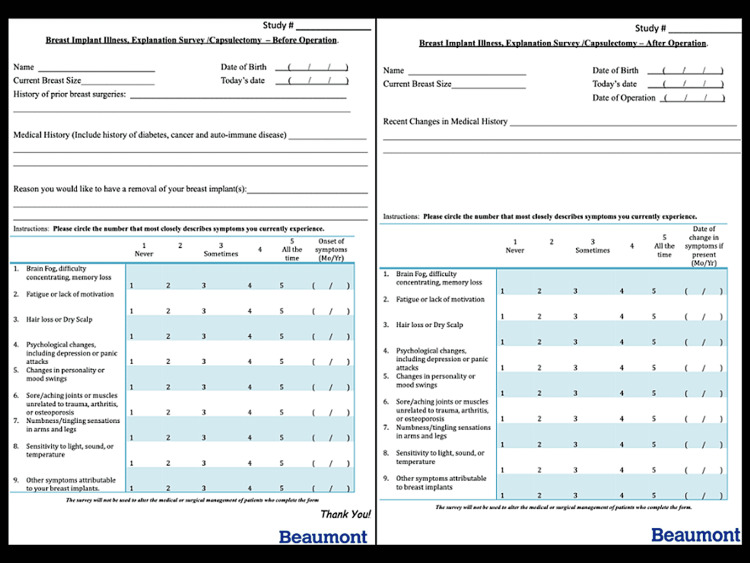
Patient survey for prospective study

The authors of this study are now collecting data in a single-center prospective study with the primary objective to correlate breast augmentation and symptomatology as well as subsequent resolution following en-bloc resection (Figure 2). Secondary endpoints seek to highlight specific symptoms appearing most commonly in women with breast implant illness and delineate which demographics are most affected. The objective is to prove that bilateral implant removal with capsulectomy is a safe and acceptable treatment for women suffering from breast implant illness.

## Conclusions

Breast implant illness is becoming more described in plastic and general surgical literature. A significant population of women currently pursue bilateral breast implant removal in hopes to alleviate this constellation of symptoms. Currently, there is no standard of care or guideline for treating women experiencing such symptoms. The current literature regarding breast implant illness has been widely observational and descriptive. With over four million women across the globe with augmented breasts, the potential impact of this research is great. This paper presents three patients believed to be suffering from breast implant illness who, after en-bloc resection, experienced resolution of their symptoms.
